# Construction and Validation of a Novel Immunosignature for Overall Survival in Uveal Melanoma

**DOI:** 10.3389/fcell.2021.710558

**Published:** 2021-09-06

**Authors:** Chufeng Gu, Xin Gu, Yujie Wang, Zhixian Yao, Chuandi Zhou

**Affiliations:** ^1^Department of Ophthalmology, Shanghai General Hospital, Shanghai Jiao Tong University School of Medicine, Shanghai, China; ^2^National Clinical Research Center for Eye Diseases, Shanghai Key Laboratory of Ocular Fundus Diseases, Shanghai Engineering Center for Visual Science and Photomedicine, Shanghai Engineering Center for Precise Diagnosis and Treatment of Eye Diseases, Shanghai, China; ^3^Department of Ophthalmology, Suzhou TCM Hospital Affiliated to Nanjing University of Chinese Medicine, Suzhou, China; ^4^Department of Urology, Shanghai General Hospital, Shanghai Jiao Tong University School of Medicine, Shanghai, China

**Keywords:** uveal melanoma, immune microenvironment, immunological marker, overall survival, prognostic signatures

## Abstract

**Objectives:**

Uveal melanoma (UM) is the most common primary intraocular malignancy in adults, and immune infiltration plays a crucial role in the prognosis of UM. This study aimed to generate an immunological marker-based predictive signature for the overall survival (OS) of UM patients.

**Methods:**

Single-sample gene-set enrichment analysis (ssGSEA) was used to profile immune cell infiltration in 79 patients with UM from The Cancer Genome Atlas (TCGA) database. Univariate and multivariate least absolute shrinkage and selection operator (LASSO) Cox regressions were used to determine the prognostic factors for UM and construct the predictive immunosignature. Receiver operating characteristic (ROC) curves, decision curve analysis (DCA), and calibration curves were performed to evaluate the clinical ability and accuracy of the model. In addition, the predictive accuracy was compared between the immunosignature and the Tumor, Node, Metastasis (TNM) staging system of American Joint Committee on Cancer (AJCC). We further analyzed the differences in clinical characteristics, immune infiltrates, immune checkpoints, and therapy sensitivity between high- and low-risk groups characterized by the prognostic model.

**Results:**

Higher levels of immune cell infiltration in UM were related to a lower survival rate. Matrix metallopeptidase 12 (MMP12), TCDD inducible poly (ADP-ribose) polymerase (TIPARP), and leucine rich repeat neuronal 3 (LRRN3) were identified as prognostic signatures, and an immunological marker-based prognostic signature was constructed with good clinical ability and accuracy. The immunosignature was developed with a concordance index (C-index) of 0.881, which is significantly better than that of the TNM staging system (*p* < 0.001). We further identified 1,762 genes with upregulated expression and 798 genes with downregulated expression in the high-risk group, and the differences between the high- and low-risk groups were mainly in immune-related processes. In addition, the expression of most of the immune checkpoint-relevant and immune activity-relevant genes was significantly higher in the high-risk group, which was more sensitive to therapy.

**Conclusion:**

We developed a novel immunosignature constructed by MMP12, TIPARP, and LRRN3 that could effectively predict the OS of UM.

## Background

Uveal melanoma (UM), arising from melanocytes in the uvea, is the most common primary intraocular malignancy in adults (Li et al., 2020). More than 90% of UM cases have choroidal involvement, and the remaining 10% are confined to the ciliary body or the iris (Shields et al., 2012). Genetic mutations in G protein subunit alpha q (GNAQ) and G protein subunit alpha 11 (GNA11) are suspected to be the initiating event in UM (Vader et al., 2017). Despite the advancements in diagnostics and therapies for UM, the prognosis of patients with UM remains unsatisfactory (Kashyap et al., 2016; Rantala et al., 2019). Almost 50% of UM patients develop metastasis and have an overall survival (OS) of less than 1 year (Kujala et al., 2003; Jager et al., 2020). Therefore, identifying high-risk patients at the initial diagnosis is of great importance and may guide clinicians in their therapeutic decisions.

The pathogenetic mechanisms and markers that influence the prognosis of UM patients have been extensively investigated in the past few decades (Li et al., 2020). Accumulating evidence reveals that chromosomal abnormalities in UM, including chromosome 3 monosomy (monosomy 3), gains of chromosomal arm 8q, and loss of chromosomal arm 1p, are closely related to an increased risk of metastasis (Aalto et al., 2001; Trolet et al., 2009; Damato et al., 2010), while gain of chromosomal arm 6p is associated with longer metastasis-free survival (MFS; Aalto et al., 2001). In addition, the genetic mutation of BRCA1-associated protein 1 (BAP1) was associated with higher chances of metastasis (Kalirai et al., 2014), while an alteration in splicing factor 3b subunit 1 (SF3B1) led to longer MFS (Yavuzyigitoglu et al., 2016). Interestingly, recent research has revealed that the gain of chromosomal 8q may worsen prognosis by activating macrophage infiltration, and the loss of BAP1 expression may drive T cell infiltration in UM (Gezgin et al., 2017), suggesting that immune infiltration plays a crucial role in the prognosis of UM.

Emerging evidence shows that the immune microenvironment is crucial for cancer progression and response to therapeutics (Quail and Joyce, 2013). In most malignancies, tumor cells escape immune responses by decreasing the expression of human leukocyte antigen (HLA; Garrido and Algarra, 2001). However, in UM, pronounced HLA expression is correlated with an increased risk of death (van Essen et al., 2016). In addition, some studies suggested that more infiltration of macrophages and CD8 + T cells in UM represents a higher risk for metastasis and poor prognosis (Whelchel et al., 1993; Bronkhorst et al., 2011), while natural killer cells play an important role in the prevention of UM metastases (Maat et al., 2009). However, the role of immune-related markers in the prognosis of UM is not fully understood.

In this study, we comprehensively profiled the immunological markers of 79 patients with UM from The Cancer Genome Atlas database (TCGA) and generated an immunological marker-based predictive signature for UM patients. We further analyzed the differences in clinical characteristics, immune infiltrates, immune checkpoints, and therapy sensitivity between high- and low-risk groups characterized by the prognostic model to investigate potential therapeutic targets.

## Materials and Methods

### Data Source

RNA-seq data and corresponding clinical information of UM samples (*n* = 79) were obtained from the TCGA^1^ as a pilot analysis. UM samples from GSE44295 (*n* = 57) and GSE22138 (*n* = 63) datasets from the Gene Expression Omnibus (GEO^2^) were used for validation.

### Immune Cell Infiltration Estimation

The relative infiltration levels of 24 immune cell types were quantified using single-sample gene-set enrichment analysis (ssGSEA) to interrogate the expression levels of gene set signatures (484 genes) based on published gene lists (Bindea et al., 2013). Then, we performed hierarchical clustering of the UM samples according to ssGSEA scores. Hierarchical clustering was performed with Euclidean distance and Ward linkage. Two distinct immune infiltration clusters, termed high infiltration and low infiltration, were defined according to the risk score. Furthermore, we analyzed the immune activity and tolerance condition of each group. We selected PD-L1, PD1, cytotoxic T-lymphocyte associated protein 4 (CTLA4), T-cell immunoglobulin mucin family member 3 (TIM3), indoleamine 2,3-dioxygenase (IDO), and lymphocyte-activation gene 3 (LAG3) as immune checkpoint-relevant signatures and CD8 antigen (CD8A), C-X-C motif chemokine ligand 10 (CXCL10), CXCL9, granzyme A (GZMA), GZMB, perforin 1 (PRF1), T-box transcription factor 2 (TBX2), and tumor necrosis factor (TNF) as immune activity-related signatures (Zhang et al., 2020).

### Survival Analysis

Kaplan–Meier survival curves were drawn using the “survival” package in R 3.5.1 to analyze the survival differences between subgroups, and the major outcome is OS, and the secondary outcome is progress-free survival (PFS). Metastasis, and tumor-related death were modeled as major outcomes (dependent factors), and patients who were alive at the end of follow-up or who died of other causes were considered censored. The survival proportions of subgroups were compared using a two-sided log-rank test.

### Prognostic Model Construction and Validation

To identify the possible correlates of the OS, 484 immunological markers of 24 immune cell types were compared using univariate Cox proportional hazards regressions. The significant factors were further applied to least absolute shrinkage and selection operator (LASSO) Cox regression analysis. The LASSO regression model analysis was performed using “glmnet” package in R 3.5.1, and non-zero coefficients and minimum of lambda were used for gene cut-off. The risk scores were estimated from the coefficients of the genes, which were calculated in accordance with the highest lambda value. Patients were stratified into the high-risk or low-risk group according to the median value of the risk score.

Receiver operating characteristic (ROC) curves and decision curve analysis (DCA) were performed to assess the clinical usefulness of the prognostic model on survival probability using the “survivalROC” and “ggDCA” packages in R 3.5.1. Higher values of area under curve (AUC) indicated better classification ability. Calibration curves of the prognostic model for the OS were examined to assess the agreement between the predicted and observed outcomes. The concordance index (C-index) of the immunosignature and the Tumor, Node, Metastasis (TNM) staging system of American Joint Committee on Cancer (AJCC) were compared using “CsChange” package in R 3.5.1.

### Functional Enrichment Analysis

Differentially expressed genes (DEGs) between the high- and low-risk groups were identified with | log2 (fold change)∣ >1.5 and adjusted *p*-value < 0.05. Gene ontology (GO) and the Kyoto Encyclopedia of Genes and Genomes (KEGG) pathway enrichment analyses of the high- and low-risk groups were performed using the “clusterprofiler” package in R 3.5.1. Gene set enrichment analysis (GSEA) was assessed the differences in signaling pathways between the high- and low-risk groups to predict the phenotypes and signaling pathways related to prognosis. The “clusterprofiler” package implements a hypergeometric test for each pathway and returns a *p*-value. A cutoff of 0.05 was used to identify enriched pathways. And the adjusted *p*-value was generated by the Bonferroni correction (“Bonferroni”) in which the *p*-values are multiplied by the number of comparisons (Benjamini and Hochberg, 1995).

### Therapeutic Sensitivity Prediction

To explore the sensitivity of therapy in the high- and low-risk groups, we predicted the drug sensitivity using the Genomics of Drug Sensitivity in Cancer (GDSC) database^3^ (Yang et al., 2013). The prediction process was implemented using the “pRRophetic” package to estimate the half maximal inhibitory concentration (IC50) of drugs by ridge regression and the prediction accuracy was evaluated by 10-fold cross-validation based on the GDSC training set (Geeleher et al., 2014).

### Statistical Analysis

All statistical analyses were performed in R 3.5.1. Mean ± standard deviation (SD) and frequency (percentage) were reported for the description of categorical variables and continuous variables. Means and proportions were compared using Student’s *t*-test, the non-parametric Mann–Whitney U test and the chi-square test (or Fisher’s exact test, if appropriate), respectively. Univariate Cox regression and LASSO Cox regression were used to determine the significant factors, and hazard ratios with 95% confidence interval (CI) were recorded. The survival and metastasis rates of subgroups were compared using a log-rank test. The comparison between immunosignature and TNM staging system was evaluated by C-index. A model with a larger C-index was considered to have a greater discrimination ability. All statistical tests were two sided, and *p* < 0.05 was considered statistically significant.

## Results

### Identification of Prognostic Signature in UM

**FIGURE 1:**
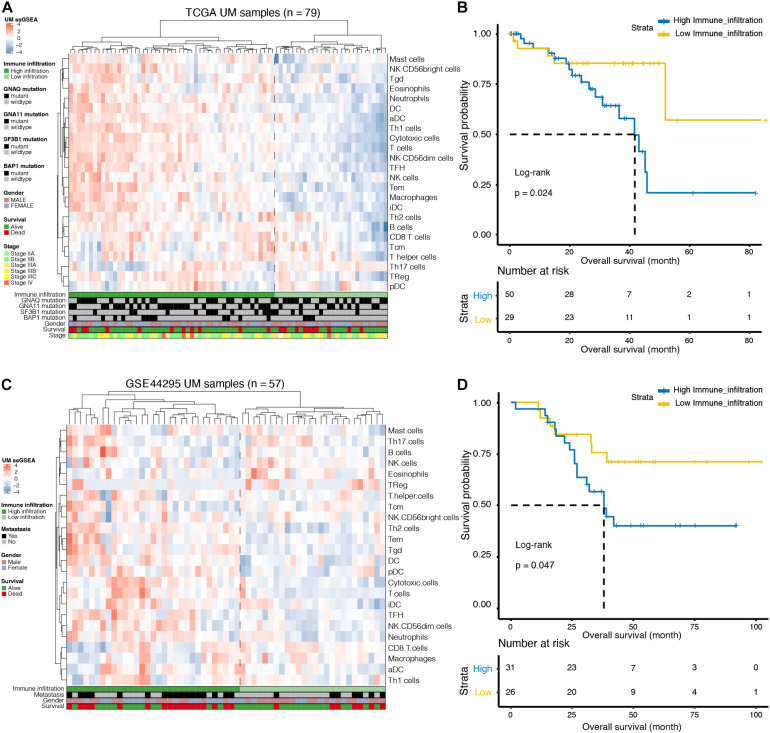
Immune landscape of uveal melanoma (UM). **(A)** Unsupervised clustering was applied to 79 UM patients from the The Cancer Genome Atlas (TCGA) database using the single-sample gene-set enrichment analysis (ssGSEA) scores of 24 immune cell types. Mutation status of G protein subunit alpha q (GNAQ), G protein subunit alpha 11 (GNA11), splicing factor 3b subunit 1 (SF3B1), and BRCA1-associated protein 1 (BAP1), sex, survival, and stage are annotated in the lower panel. **(B)** Kaplan–Meier curves for the overall survival (OS) of UM patients in the TCGA dataset grouped by high and low infiltration levels. **(C)** Unsupervised clustering was applied to 57 UM patients from the GSE44295 dataset using the ssGSEA scores of 24 immune cell types. Metastatic status, sex, and survival are annotated in the lower panel. **(D)** Kaplan–Meier curves for the OS of UM patients in the GSE44295 dataset grouped by high and low infiltration levels.

We used ssGSEA to quantify 24 immune cell types in the TCGA cohort and noted heterogeneity in the infiltration of immune cell types in UM patients (Figure 1A). Through hierarchical clustering, we found two distinct groups with different immune infiltration patterns. Mutation status of GNAQ, GNA11, SF3B1, and BAP1, sex, survival, and clinical stage are annotated in the lower panel (Figure 1A). Further, the Kaplan–Meier curve showed that the cumulative survival rate of patients with high infiltration status was significantly lower than that of patients in the low infiltration group (*p* = 0.024, Figure 1B). Similarly, this phenomenon was further validated in the GSE44295 cohort: infiltration heterogeneity also appeared in UM patients (Figure 1C), and patients with high infiltration status showed lower OS (*p* = 0.047, Figure 1D).

**FIGURE 2:**
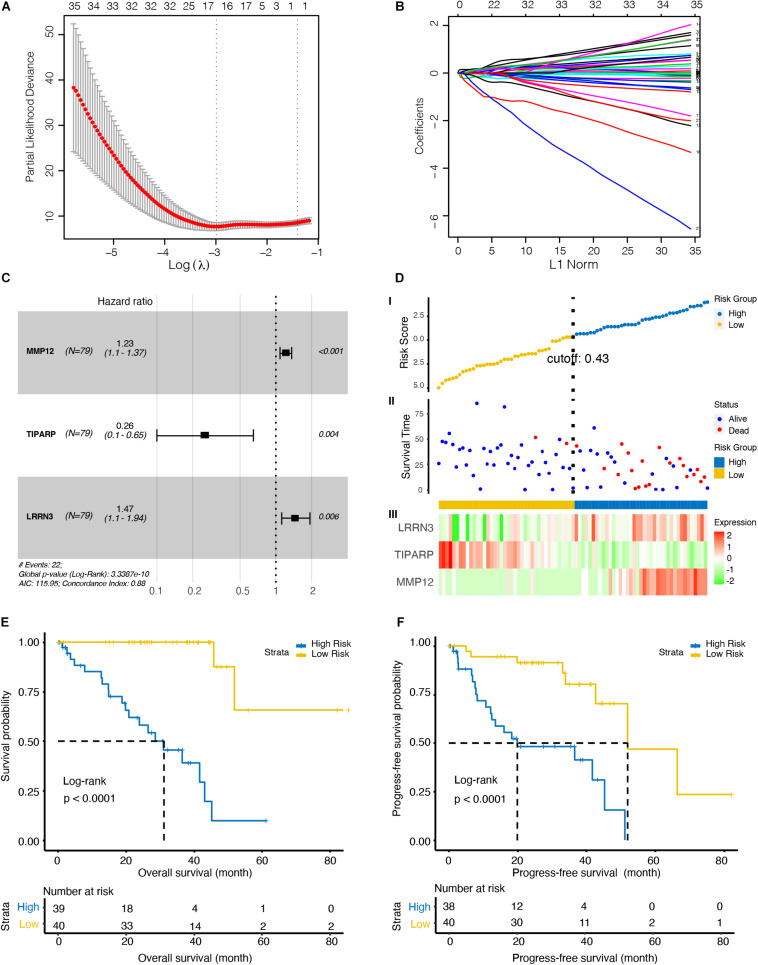
Identification of an optimal marker model for prognostic prediction in the TCGA dataset UM patients. **(A)** 10-fold cross-validation for tuning parameter selection in the least absolute shrinkage and selection operator (LASSO) model. The first vertical line equals the minimum error (lambda = 0.05265), whereas the second vertical line shows the cross-validated error within one standard error of the minimum (lambda = 0.17646). **(B)** LASSO coefficient profiles of the fractions of 219 immune cell markers. Each curve corresponds to a variable. It shows the path of its coefficient against the L1-Norm of the whole coefficient vector as lambda varies. The axis above indicates the number of non-zero coefficients at the current lambda, which is the effective degrees of freedom (df) for the LASSO. **(C)** Forest plots showing associations between different immune cell markers and OS in the TCGA cohort. Unadjusted hazard ratios are shown with 95% confidence interval (CI). **(D)** Characteristics of the 3-gene pair prognostic signature. (top): the risk score of each UM patient; (middle): OS and survival status of UM patients; (bottom): heat map of gene expression profiles of UM patients in the TCGA cohort. **(E)** Kaplan–Meier curves for the OS of UM patients in high- and low-risk groups. **(F)** Kaplan–Meier curves for the progress-free survival (PFS) of UM patients in high- and low-risk groups.

To further investigate the immune-related biomarkers that could predict the prognosis of UM, univariate Cox regression and LASSO Cox regression were used to analyze the roles of 484 immunological markers of 24 immune cells. After univariate Cox regression analyses, 219 factors were further analyzed by LASSO Cox regression. After 10-fold cross-validation, we used non-zero coefficients for gene cut-off and the minimum criteria for lambda was 0.17646, which is the value of lambda that gives the most regularized model such that the cross-validated error is within one standard error of the minimum (Figures 2A,B and Supplementary Figure 1). Three genes were identified: matrix metallopeptidase 12 (MMP12), TCDD inducible poly (ADP-ribose) polymerase (TIPARP), and leucine rich repeat neuronal 3 (LRRN3). The hazard ratio of MMP12, TIPARP, and LRRN3 was 1.23 (95% CI, 1.1–1.37), 0.26 (95% CI, 0.1–0.65), and 1.47 (95% CI, 1.1–1.94), respectively (Figure 2C). Then, we constructed a risk signature based on the expression of specific genes [transcripts per kilobase of exon model per million mapped reads (TPM)] and the coefficients from the Cox regression. The risk score formula was calculated as follows:

Riskscore=0.20332×(TPMofMMP12)-1.36517×(TPMofTIPARP)+0.38766×(TPMofLRRN3)

To explore the optimal cutoff value that could be used to stratify patients into the high- and low-risk groups, the time-dependent ROC curve analysis was used, and the optimal cutoff value was determined to be 0.43 (Figure 2D). The heat map showed the expression level of the three genes in each UM patient (Figure 2D). We further investigate the prognosis of patients in high- and low-risk groups characterized by the prognostic model. Kaplan–Meier curves revealed that patients in the high-risk group had lower OS (*p* < 0.001, Figure 2E) and PFS (*p* < 0.001, Figure 2F) than patients in the low-risk group.

### Validation of Prognostic Signature in UM

**FIGURE 3:**
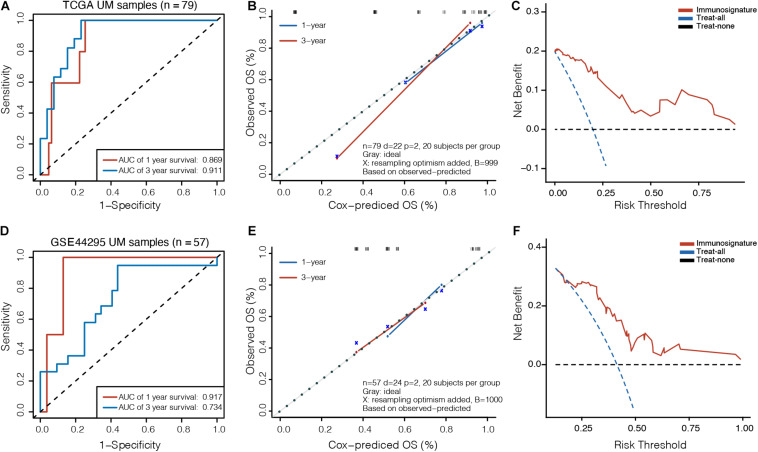
Validation of prognostic model in UM. **(A)** Time-dependent receiver operating characteristic (ROC) curves, **(B)** calibration plots, and **(C)** decision curve analysis (DCA) for the OS predicted with the prognostic model in the TCGA cohort. **(D)** Time-dependent ROC curves, **(E)** calibration plots, and **(F)** DCA for the OS predicted with the prognostic model in the GSE44295 cohort. The *x*-axis of calibration plot is the immunosignature-predicted probability of OS, and the *y*-axis is the actual OS.

To assess the clinical usefulness of the prognostic model on the survival probability of UM patients, ROC, calibration plots, and DCA curves were performed. The AUC values for the 1− and 3-year OS in the TCGA dataset were 0.869 (95% CI, 0.767–0.971) and 0.911 (95% CI, 0.827–0.995), respectively (Figure 3A). The calibration plots displayed fair agreement between the predictions and actual observations for the 1− and 3-year OS in the TCGA dataset (Figure 3B), and the DCA showed that using the prognostic signature to predict the OS had more benefit than either the “treat-all” model or the “treat-none” model for most of patients in the TCGA dataset (Figure 3C). In the GSE44295 validation dataset, AUC values of 0.917 (95% CI, 0.827–1.000) and 0.734 (95% CI, 0.592–0.877) were obtained for the 1− and 3-year OS, respectively (Figure 3D). The calibration plots displayed favorable agreement between the predictions and actual observations (Figure 3E), and the DCA showed that using the prognostic signature to predict the OS was more beneficial for most of patients in the GSE44295 dataset (Figure 3F). The prognostic signature has also been proven effective in the GSE22138 validation dataset (Supplementary Figure 2).

We further compared the accuracy of the immunosignature with the conventional TNM staging system. The immunosignature has a more stable discrimination ability than TNM staging system (Supplementary Figure 3). The C-index of the immunosignature was 0.881 (95% CI, 0.823–0.939), whereas for TNM staging, the C-index was limited to 0.619 (95%CI, 0.482–0.756). The details of the comparisons are summarized in Table 1.

**TABLE 1:** The comparison of predictive discrimination ability of the immunosignature and Tumor, Node, Metastasis (TNM) staging system.

	C-index (95% CI)	*p*-value	Comparison of models	
			C-index (95% CI)	*p*-value
Immunosignature	0.881 (0.823, 0.939)	<0.001*	–	–
T stage	0.619 (0.482, 0.756)	0.090	−0.262 (−0.417, −0.137)	<0.001*

T, Tumor category according to 8th edition of American Joint Committee on Cancer (AJCC) staging system; CI, confidence interval; *Statistically significant.

### The Comparisons of Clinical and Pathologic Characteristics of Patients Between High- and Low-Risk Groups

Furthermore, we investigated the clinical and pathologic characteristics of patients in high- and low-risk groups. There were no significant differences in age (*p* = 0.148) or sex (*p* = 1.000) between the high- and low-risk groups. More than half of the patients in the high-risk group were in stage III or IV, while 60% of the patients in the low-risk group were in stage II (*p* = 0.038). Moreover, in terms of histological type, patients in the high-risk group mainly had more epithelioid cell type than those in the low-risk group (*p* = 0.011). In addition, the status of chromosomes 3, 8q, 6p, and 1p in the different subgroups was assessed. The proportion of monosomy 3 accounted for 85% in the high-risk group but only 12% in the low-risk group (*p* < 0.001). The gains of 8q and 6p were 79 and 26%, respectively, in the high-risk group, while the proportions in the low-risk group were 35 and 78% (all *p* < 0.001). Furthermore, the mutation status of SF3B1, BAP1, GNAQ, and GNA11 in the different risk subgroups was assessed. The mutation of SF3B1 was significantly higher in the low-risk group (*p* = 0.004). The clinical and pathologic characteristics of patients are detailed in Table 2.

**TABLE 2:** Clinical and pathologic characteristics of patients in the high- and low-risk groups.

Variables	Total (*n* = 79)	High risk (*n* = 39)	Low risk (*n* = 40)	*p*-value
**Age (years)**	61.47 ± 13.94	63.77 ± 13.11	59.23 ± 14.52	0.148
**Gender**				1.000
Female	35(44%)	17(44%)	18(45%)	
Male	44(56%)	22(56%)	22(55%)	
**T Stage**				0.038*
Stage II	39(49%)	15(38%)	24(60%)	
Stage III	35(44%)	19(49%)	16(40%)	
Stage IV	4(5%)	4(10%)	0(0%)	
Missing	1(1%)	1(3%)	0(0%)	
**Histological type**				0.011*
Epithelioid cell type	13(16%)	10(26%)	3(8%)	
Spindle cell type	30(38%)	9(23%)	21(52%)	
Mixed cell type	36(46%)	20(51%)	16(40%)	
**Chromosome 3 status**				< 0.001*
Monosomy 3	38(48%)	33(85%)	5(12%)	
Disomy 3	39(49%)	5(13%)	34(85%)	
Missing	2(3%)	1(3%)	1(2%)	
**Chromosome 8q status**				< 0.001*
Gained	45(57%)	31(79%)	14(35%)	
Not called	21(27%)	5(13%)	16(40%)	
Missing	13(16%)	3(8%)	10(25%)	
**Chromosome 6p status**				< 0.001*
Gained	41(52%)	10(26%)	31(78%)	
Not called	36(46%)	28(72%)	8(20%)	
Missing	2(3%)	1(3%)	1(2%)	
**Chromosome 1p status**				0.184
Lost	17(22%)	11(28%)	6(15%)	
Not called	57(72%)	27(69%)	30(75%)	
Missing	5(6%)	1(3%)	4(10%)	
**SF3B1 status**				0.004*
Mutant	18(23%)	3(8%)	15(38%)	
Wild type	61(77%)	36(92%)	25(62%)	
**BAP1 status**				0.076
Mutant	15(19%)	11(28%)	4(10%)	
Wild type	64(81%)	28(72%)	36(90%)	
**GNAQ status**				0.055
Mutant	38(48%)	14(36%)	24(60%)	
Wild type	41(52%)	25(64%)	16(40%)	
**GNA11 status**				0.056
Mutant	35(44%)	22(56%)	13(32%)	
Wild type	44(56%)	17(44%)	27(68%)	

*T, Tumor category according to 8th edition of American Joint Committee on Cancer (AJCC) staging system; SF3B1, splicing factor 3b subunit 1; BAP1, BRCA1-associated protein 1; GNAQ, G protein subunit alpha q; GNA11, G protein subunit alpha 11.*

Data are presented as mean ± standard deviation (SD)/*n* (%). *Statistically significant.

### Functional Annotation Between High- and Low-Risk Groups of UM Patients

**FIGURE 4:**
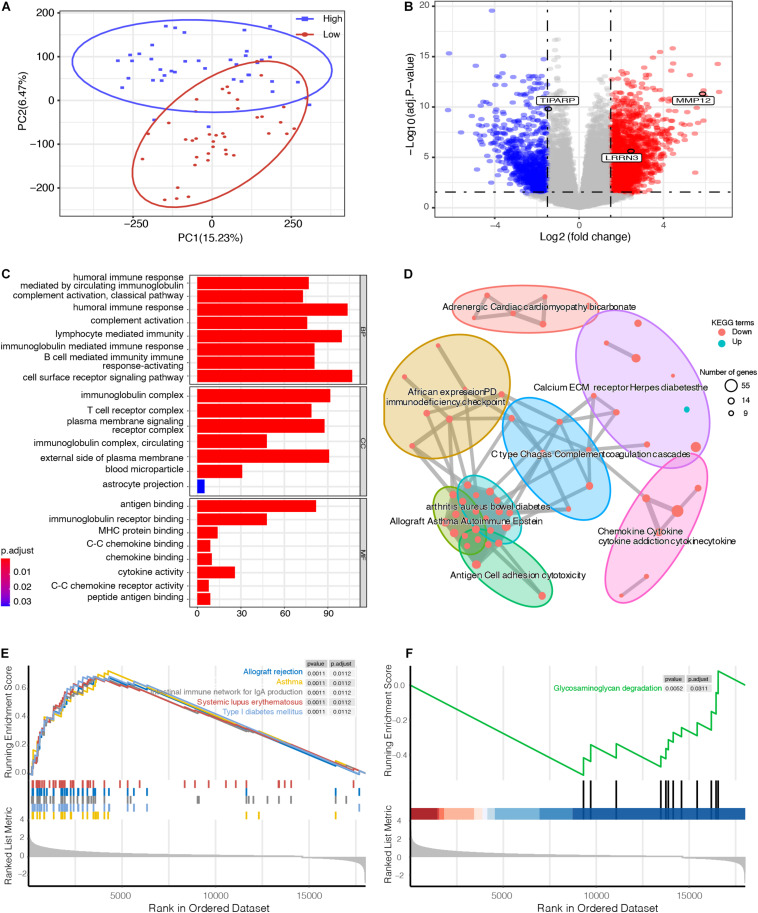
Functional enrichment analysis between high- and low-risk UM groups. **(A)** principal component analysis (PCA) successfully separated the high- and low-risk UM patients. **(B)** Volcano plot displaying the differentially expressed genes (DEGs) between the high- and low-risk groups. The log2 (fold change) of TCDD inducible poly (ADP-ribose) polymerase (TIPARP), leucine rich repeat neuronal 3 (LRRN3), and matrix metallopeptidase 12 (MMP12) was –1.46, 2.46, and 5.86, respectively. **(C)** Gene ontology (GO), **(D)** Kyoto Encyclopedia of Genes and Genomes (KEGG), and **(E,F)** Gene set enrichment analysis (GSEA) analyses compared the difference between the high- and low-risk UM groups.

To explore the underlying biological mechanisms of distinct immunophenotypes, we performed differential and functional analyses to identify DEGs and pathways between the high- and low-risk groups. The result of principal component analysis (PCA) was consistent with the risk classification (Figure 4A). We identified 1,762 genes with upregulated expression and 798 genes with downregulated expression in the high-risk group (Figure 4B and Supplementary Table 1). And GO analysis, including biological process (BP), cellular component (CC), and molecular function (MF), and KEGG functional enrichment analysis were further performed. The GO analysis revealed that the DEGs were mostly enriched in immune responses, such as the humoral immune response, lymphocyte mediated immunity, and the T cell receptor complex (Figure 4C). Moreover, KEGG analysis also revealed that DEGs were significantly enriched in immunity-related pathways (Figure 4D), such as Cytokine–cytokine receptor interaction, Graft-versus-host disease, and Allograft rejection (Supplementary Figure 4). A detailed table of KEGG terms is provided in Supplementary Table 2. Besides, GSEA analysis also revealed that the DEGs were mostly enriched in immune responses, including Allograft rejection, Asthma, and Intestinal immune network for IgA production (Figures 4E,F).

**FIGURE 5:**
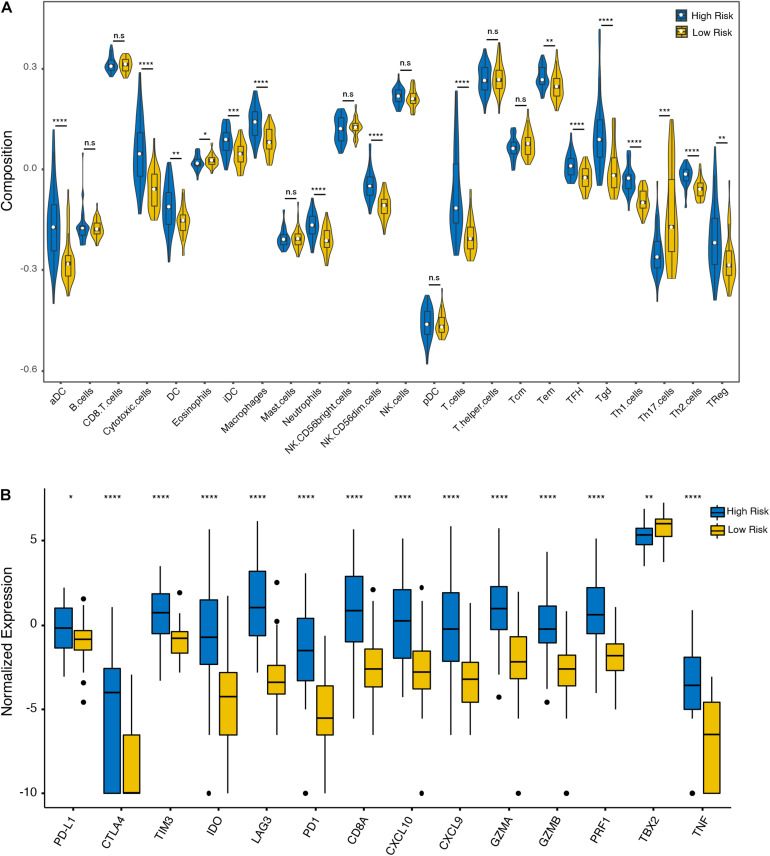
Immune cell and checkpoint analysis between the high- and low-risk UM groups. **(A)** Differential proportions of immune cells between high- and low-risk groups in the TCGA cohort. **(B)** Differential expression of immune checkpoint immune checkpoints between the high- and low-risk groups in the TCGA cohort. *p < 0.05; **p < 0.01; ***p < 0.001; ****p < 0.0001; and n.s, no significant.

To further analyze the immune activity and tolerance condition of each group, the variations of immune cells and checkpoints between the high- and low-risk groups were investigated. In general, the degree of cell infiltration was lower in the low-risk group than in the high-risk group, except for eosinophils and T helper 17 (Th17) cells (Figure 5A). In addition, we found that most of the immune checkpoint-relevant and immune activity-relevant genes were significantly higher in the high-risk group, except for TBX2 (Figure 5B).

### High-Risk Group Was More Sensitive to Therapy

**FIGURE 6:**
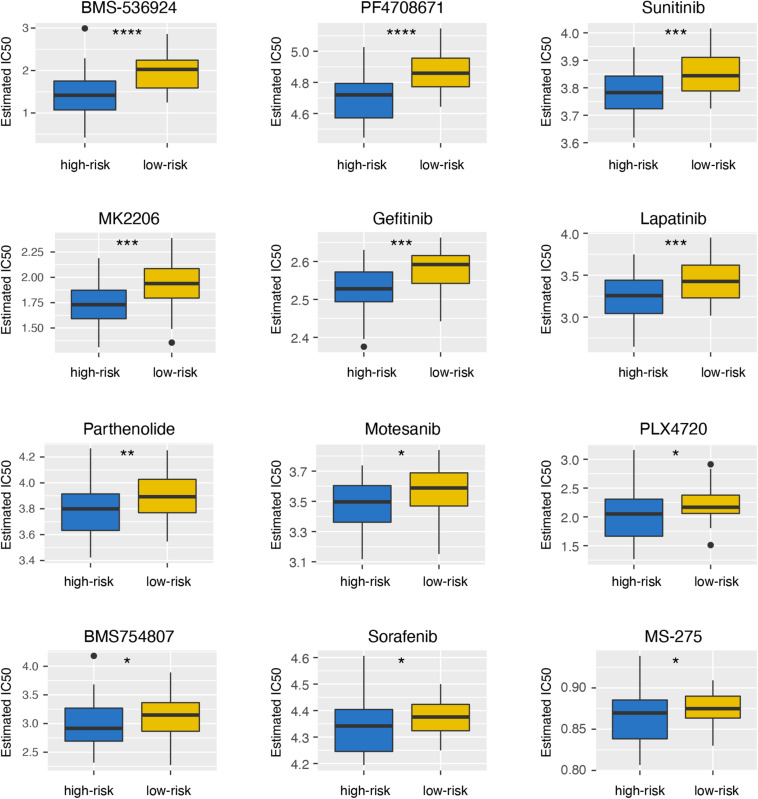
Differential sensitivity to drugs between the high- and low-risk groups. *p < 0.05; **p < 0.01; ***p < 0.001; and ****p < 0.0001.

To explore the differences of drug sensitivity between the high- and low-risk groups, we used GDSC database to estimate the IC50 of drugs. Twelve therapeutic drugs were identified, including BMS-536924, PF4708671, Sunitinib, MK2206, Gefitinib, Lapatinib, Parthenolide, Motesanib, PLX4720, BMS754807, Sorafenib, and MS-275, as the estimated IC50 of these chemotherapeutic drugs were lower in the high-risk group than those in the low-risk group (Figure 6).

## Discussion

In the present study, we initially identified MMP12, TIPARP, and LRRN3 as crucial immunological markers related to the prognosis of UM and generated an immunological marker-based predictive signature for UM patients. MMP12 is one of the immunological markers of immature dendritic cells (iDCs), and this gene is located on chromosome 11 at 11q22.2. MMP12 is an elastolytic MMP and capable of degrading extracellular matrix components (Chelluboina et al., 2018). MMP12 plays a pivotal role in inflammatory diseases, such as colitis (Nighot et al., 2021) and chronic obstructive pulmonary disease (Baggio et al., 2020). In addition, MMP12 has been regarded as a potential prognostic biomarker for ovarian cancer (Guo et al., 2020) and gallbladder cancer (Zhao et al., 2019). Our results initially identified MMP12 as a negative prognostic biomarker for UM. TIPARP is one of the immunological markers of eosinophils, and its gene is located on chromosome 3 at 3q25.31. TIPARP is a transcriptional repressor of aryl hydrocarbon receptor (AHR; MacPherson et al., 2013), which plays an important role in the immune system (Stevens et al., 2009). We firstly identified TIPARP as a favorable prognostic biomarker for the OS of UM. In line with our results, higher TIPARP expression has been reported to be related to better survival in breast cancer (Cheng et al., 2019). LRRN3 is one of the immunological markers of Th1 cells, and this gene is located on chromosome 7 at 7q31.1. Chou et al. (2013) found that reduced LRRN3 gene expression correlates with the senescent phenotype of CD8 + T cells. In neuroblastomas, low expression of LRRN3 is associated with a lower survival rate (Akter et al., 2011). In our study, LRRN3 is a negative prognostic biomarker for OS in UM.

Accumulating evidence shows that monosomy 3 and gain of chromosomal arm 8q are closely related to an increased risk of metastasis (Aalto et al., 2001; Trolet et al., 2009; Damato et al., 2010), while gain of chromosomal arm 6p and genetic alteration of SF3B1 correlate with longer MFS (Aalto et al., 2001; Yavuzyigitoglu et al., 2016). Consistent with the results of previous studies, the high-risk group characterized by the immunological marker-based prognostic model mainly had more epithelioid cell type, monosomy 3, 8q gains, but less 6p gains, and mutant in SF3B1. These clinical characteristics further prove that this immunological prognostic model could complement the molecular mechanism of UM.

It is well known that the eye is considered an immune privileged region (Forrester and Xu, 2012). There are many soluble immune suppressors in the aqueous humor, including transforming growth factor-beta (TGF-β) (Wilbanks et al., 1992), macrophage migration inhibitory factor (MIF; Apte et al., 1998), IDO, and CTLA (Li et al., 2020). In contrast to many other malignancies, the presence of an immune infiltrate in UM is associated with a poor prognosis (Bronkhorst et al., 2012), which is consistent with our findings. However, our results also suggested that eosinophils and Th17 cells were associated with better OS. Consistently, Heppt et al. (2017) found that a relative eosinophil count <1.5% was an independent risk factor for poor survival in UM. Th17 cells are known to characteristically secrete IL-17A, IL-17F, and IL-22 (Tian et al., 2021). Martin-Orozco et al. (2009) found that IL-17A-deficient mice were more susceptible to develop lung melanoma, suggesting that Th17 cells may exert the protective effect from metastasis. These results confirmed that the immune response played an important role in the prognosis of UM patients. However, to date, the efficacy of immune checkpoint blockade in UM is poorer than that in cutaneous melanoma (Li et al., 2020). In our study, we found that the expression of immune checkpoint-relevant genes, including PD-L1, PD1, CTLA4, TIM3, IDO, and LAG3, was significantly higher in the high-risk group, which suggests that patients in the high-risk group may be more sensitive to immunotherapies. TBX2 is a member of the T-box family of transcription factors, which is associated with a poor prognosis in multiple tumors, such as gastric (Lu et al., 2020), breast (Wang et al., 2012), and colorectal (Han et al., 2013) cancers. In addition, Wansleben et al. (2013) found that TBX2 overexpression was related to chemotherapeutic drug resistance and that targeting TBX2 could improve the efficacy of anticancer treatments. However, no studies have been reported on the expression of TBX2 in UM patients. Our study revealed that the expression of TBX2 was higher in the low-risk group of UM patients, suggesting that targeting TBX2 in combination with anticancer drugs may be an effective treatment for patients in the low-risk group. The selective use of immune checkpoint blockade may be a future direction of UM management, and our study provides the basis for an exploration of precision medicine approaches.

Numerous studies suggest that calcium homeostasis dysfunction may be strongly associated with the malignant and metastatic phenotype of UM (Li et al., 2019; Piaggio et al., 2019). S100, a calcium-binding protein, plays an important role in the development of UM (Keijser et al., 2006). Our study revealed that the expression of S100A1, S100A2, S100A3, S100A4, and S100A6 was significantly upregulated in the high-risk subgroup than the low-risk group (Supplementary Figure 5), suggesting that immune infiltration may influence UM development by regulating calcium homeostasis. However, further investigations are required to explore the mechanisms underlying these associations.

In this study, we developed a novel immunosignature constructed by MMP12, TIPARP, and LRRN3 that could effectively predict the OS of UM. In addition, our results suggested that eosinophils and Th17 cells may have a protective effect on the prognosis of UM, and calcium homeostasis may play an important role in immune infiltration. Future studies and clinical trials are warranted to further validate our findings.

## Data Availability Statement

RNA-seq data and corresponding clinical information were acquired from the TCGA database (https://portal.gdc.cancer.gov/projects/TCGA-UVM), the GSE44295 dataset (https://www.ncbi.nlm.nih.gov/geo/query/acc.cgi?acc=GSE44295), and the GSE22138 dataset (https://www.ncbi.nlm.nih.gov/geo/query/acc.cgi?acc=GSE22138) in the GEO.

## Author Contributions

CG: conceptualization, methodology, data curation, writing—original draft and review and editing, and visualization. XG: conceptualization, methodology, data curation, writing—original draft and review and editing. YW: conceptualization, software, formal analysis, writing—original draft, and visualization. ZY: conceptualization, writing—review and editing, visualization, supervision, and project administration. CZ: conceptualization, writing—review and editing, supervision, project administration, and funding acquisition. All authors contributed to the article and approved the submitted version.

## Conflict of Interest

The authors declare that the research was conducted in the absence of any commercial or financial relationships that could be construed as a potential conflict of interest.

## Publisher’s Note

All claims expressed in this article are solely those of the authors and do not necessarily represent those of their affiliated organizations, or those of the publisher, the editors and the reviewers. Any product that may be evaluated in this article, or claim that may be made by its manufacturer, is not guaranteed or endorsed by the publisher.

## Footnotes

1
https://portal.gdc.cancer.gov/projects/TCGA-UVM

2
https://www.ncbi.nlm.nih.gov/geo/

3
https://www.cancerrxgene.org
