# Supplementary material for: Construction and Validation of a Novel Immunosignature for Overall Survival in Uveal Melanoma
Source: Front Cell Dev Biol. 2021 Sep 6;9:710558. doi: 10.3389/fcell.2021.710558 (PMC8450517; doi:10.3389/fcell.2021.710558)
Supplement: Supplementary file 1 [file Data_Sheet_1.pdf]

Supplementary Material

1. Supplementary Figures

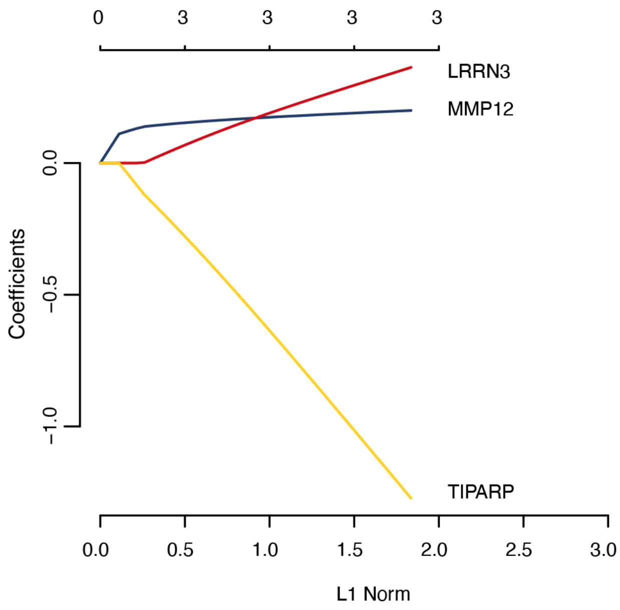

Supplementary Figure 1. LASSO Cox model fitting curve with a threshold of lambda = 0.176464. Each curve corresponds to a variable. It shows the path of its coefficient against the L1-Norm of the whole coefficient vector as lambda varies. The axis above indicates the number of non-zero coefficients at the current lambda, which is the effective degrees of freedom (df) for the LASSO.

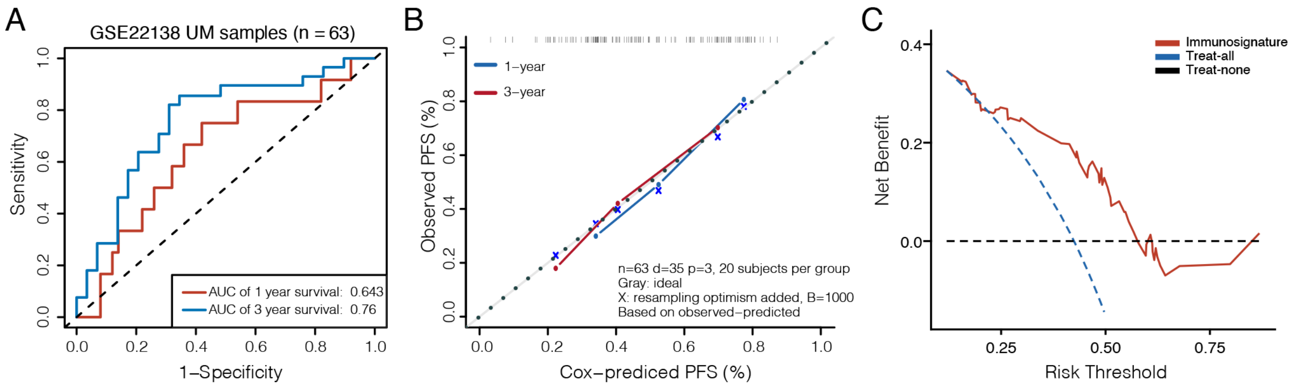

Supplementary Figure 2. Validation of prognostic model in UM. (A) Time-dependent receiver operating characteristic (ROC) curves, (B) calibration plots, and (C) decision curve analysis for the progress-free survival (PFS) predicted with the prognostic model in the GSE22138 cohort. The AUC values for the 1-year and 3-year OS in the GSE22138 cohort were 0.643 (95% CI, 0.450 to 0.837) and 0.76 (95% CI, 0.617 to 0.903), respectively. The calibration plots displayed fair agreement between the predictions and actual observations for the 1-year and 3-year PFS in the GSE22138 cohort, and the DCA showed that using the prognostic signature to predict the PFS had more benefit than the "treat-all" model for most of patients in the GSE22138 cohort.

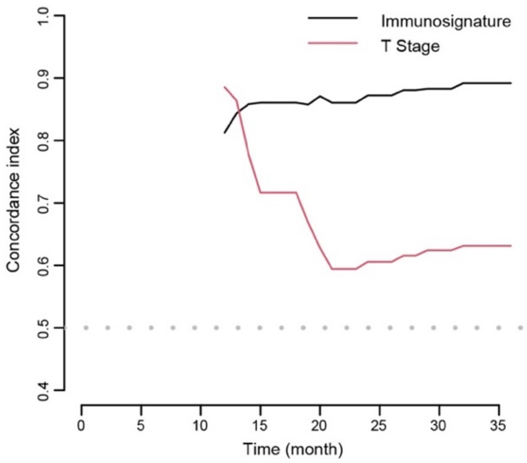

Supplementary Figure 3. The predictive discrimination ability of the immunosignature and Tumor, Node, Metastasis (TNM) staging system. The immunosignature has a more stable discrimination ability than TNM staging system.

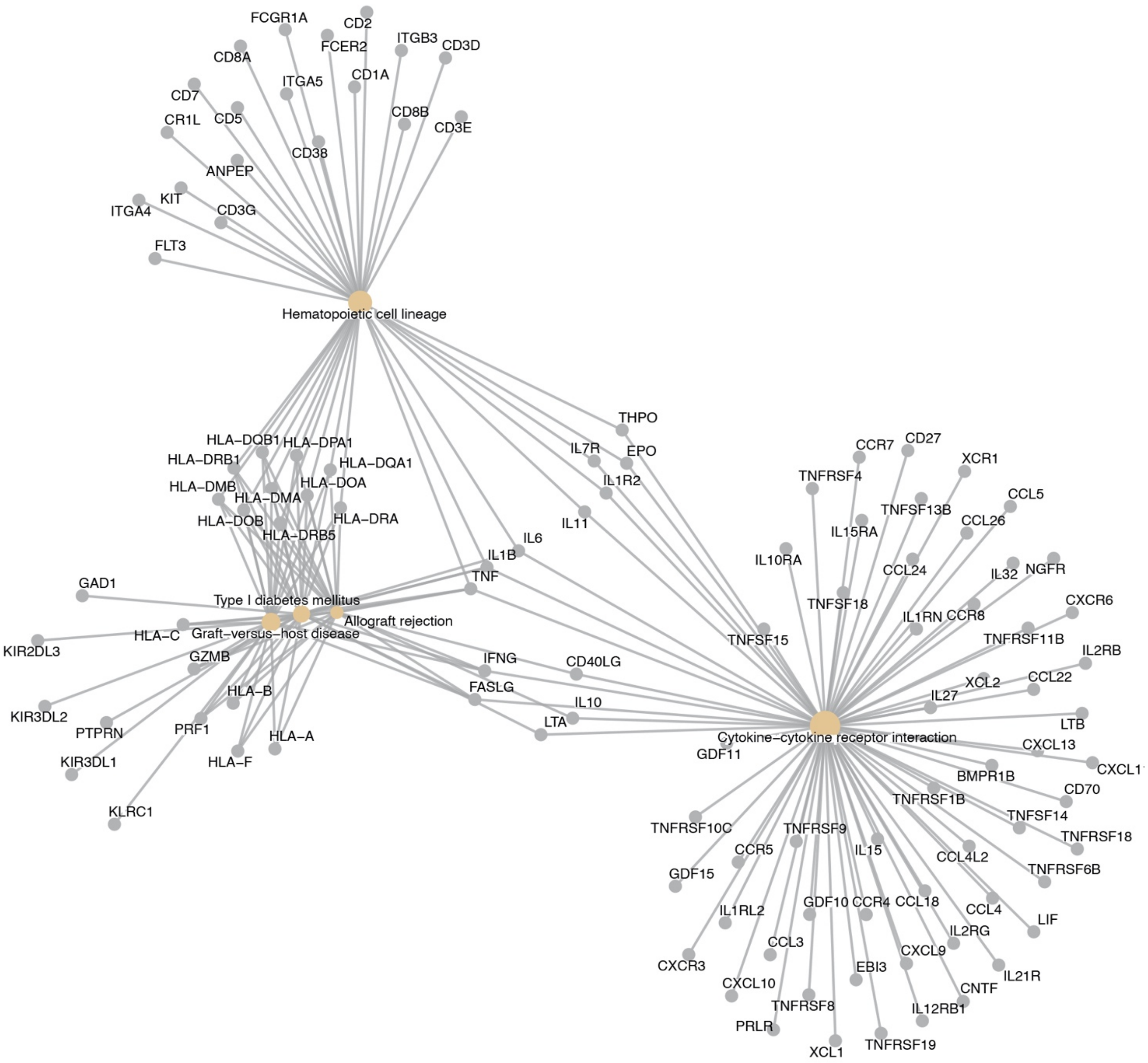

Supplementary Figure 4. The gene-gene interactions between KEGG pathways of Cytokine-cytokine receptor interaction, Graft-versus-host disease, Allograft rejection, Hematopoietic cell lineage, and Type I diabetes mellitus.

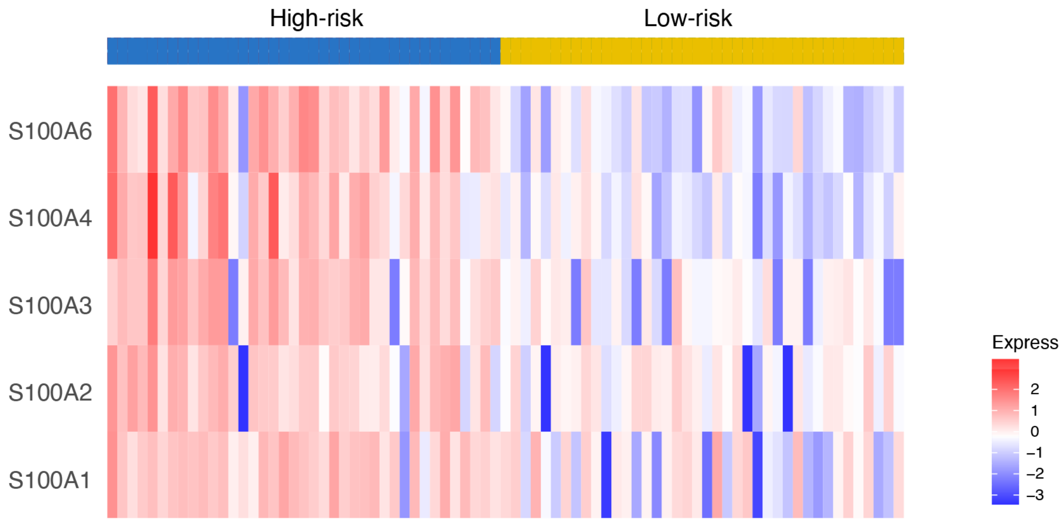

Supplementary Figure 5. The heat map of S100A1, S100A2, S100A3, S100A4, and S100A6 in high- and low-risk groups. The expression of S100A1, S100A2, S100A3, S100A4, and S100A6 was significantly upregulated in the high-risk subgroup than the low-risk group.
